# Ginsenoside Rb1 enhances atherosclerotic plaque stability by skewing macrophages to the M2 phenotype

**DOI:** 10.1111/jcmm.13329

**Published:** 2017-09-25

**Authors:** Xue Zhang, Ming‐hao Liu, Lei Qiao, Xin‐yu Zhang, Xiao‐ling Liu, Mei Dong, Hong‐yan Dai, Mei Ni, Xiao‐rong Luan, Jun Guan, Hui‐xia Lu

**Affiliations:** ^1^ The Key Laboratory of Cardiovascular Remodeling and Function Research Chinese Ministry of Education and Chinese Ministry of Health; The State and Shandong Province Joint Key Laboratory of Translational Cardiovascular Medicine; Department of Cardiology Qilu Hospital of Shandong University Jinan China; ^2^ Department of Cardiology Qingdao Municipal Hospital Qingdao China

**Keywords:** Ginsenoside Rb1, M2 macrophages, inflammation, atherosclerotic plaque stability

## Abstract

Atherosclerosis (AS) is characterized as progressive arterial plaque, which is easy to rupture under low stability. Macrophage polarization and inflammation response plays an important role in regulating plaque stability. Ginsenoside Rb1 (Rb1), one of the main active principles of *Panax Ginseng*, has been found powerful potential in alleviating inflammatory response. However, whether Rb1 could exert protective effects on AS plaque stability remains unclear. This study investigated the role of Rb1 on macrophage polarization and atherosclerotic plaque stability using primary peritoneal macrophages isolated from C57BL/6 mice and AS model in ApoE^−/−^ mice. *In vitro*, Rb1 treatment promoted the expression of arginase‐I (Arg‐I) and macrophage mannose receptor (CD206), two classic M2 macrophages markers, while the expression of iNOS (M1 macrophages) was decreased. Rb1 increased interleukin‐4 (IL‐4) and interleukin‐13 (IL‐13) secretion in supernatant and promoted STAT6 phosphorylation. IL‐4 and/or IL‐13 neutralizing antibodies and leflunomide, a STAT6 inhibitor attenuated the up‐regulation of M2 markers induced by Rb1. *In vivo*, the administration of Rb1 promoted atherosclerotic lesion stability, accompanied by increased M2 macrophage phenotype and reduced MMP‐9 staining. These data suggested that Rb1 enhanced atherosclerotic plaque stability through promoting anti‐inflammatory M2 macrophage polarization, which is achieved partly by increasing the production of IL‐4 and/or IL‐13 and STAT6 phosphorylation. Our study provides new evidence for possibility of Rb1 in prevention and treatment of atherosclerosis.

## Introduction

Coronary artery disease (CAD) has become the global health problem in both developed and developing countries. Atherosclerotic plaque has been proved pathological basis. With progressive growth of atherosclerosis (AS), plaques become vulnerable and are easy to rupture 1, 2. Efforts to understand the dynamic change in plaque help us seek for new therapy to stabilize the AS plaque 3, 4.

It is well established that macrophages play a pivotal role in the whole AS progression *via* infiltration and switch of population, which has been termed as ‘macrophage polarization’ in recent literatures 5, 6. Macrophage can differentiate into two antagonistic subtypes: classically activated by interferon‐gamma (IFN‐ɣ) or lipopolysaccharide (LPS), known as M1; or alternatively activated by interleukin (IL) ‐13 or IL‐4, known as M2 7. The two phenotypes of macrophage play a different role in inflammation through production of pro‐inflammatory or anti‐inflammatory cytokines, depending on micro‐environmental stimuli 8, 9, 10, 11. Pro‐inflammatory M1 macrophages could lead to a more vulnerable plaque while anti‐inflammatory M2 macrophages have protective effects 12. Therefore, promoting M2 macrophage polarization may provide a new approach to alleviate inflammation and increase AS plaque stability.

*Panax Ginseng* has been proved one of the greatest medical ingredients in traditional Chinese medicine through thousands of years. Active components of ginseng have been separated and identified recently. Ginsenoside Rb1 is one of the main active components derived from ginseng 13. The pharmacological effects of Rb1 have been determined in recent years 14. Apart from a series of benefits, Rb1 also shows powerful vascular‐protective potential 13, 14, 15, but its effect on atherosclerosis and detailed mechanisms needs further elucidated. Considering the important role of macrophage polarization in inflammation and atherosclerosis, we propose the hypothesis that Rb1 may protect against atherosclerosis by skewing macrophage to the anti‐inflammatory M2 phenotype.

To test the hypothesis, we cultured and stimulated peritoneal macrophages and RAW 264.7 cells with Rb1 to search for possible pathway *in vitro*. We also validated the anti‐atherosclerosis effect in atherosclerotic ApoE^−/−^ mice with Rb1 treatment *in vivo*.

## Materials and methods

### Ethics approval

The experiment protocols complied with the Animal Management Rules of the Chinese Ministry of Health (document no. 55, 2001) and conform to the NIH guidelines (the Guide for the Care and Use of Laboratory Animals published by the National Institutes of Health; NIH Publication No. 85‐23, revised 1996). Ethics committee of Qilu Hospital of Shandong University has approved all the animal experiments.

### Isolation of peritoneal macrophages

C57BL/6 mice (8‐10 weeks old) were injected intraperitoneally with 1 ml 3% Thioglycollate Broth (Fluka, Shanghai, China) to recruit macrophages to peritoneal cavity according to previous study 16. Three days post‐injection, peritoneal macrophages were collected by mouse peritoneal cavity using cold sterile PBS. The cell pellets were obtained after centrifugation at 800 r.p.m. for 5 min. The cells were incubated in 1640 RPMI (Beyotime Biotechnology, Shanghai, China) supplemented with 10% FBS (Gibco®, ThermoFisher Scientific, Shanghai, China) and 1% antibiotics (Gibco®) for 3 hrs and washed three times to remove non‐adherent cells.

### Cell culture and treatment

Peritoneal macrophages were pre‐treated with indicated concentrations of Ginsenoside Rb1 (Fleton Natural Products, Chengdu, Sichuan, China) for 1 h prior to the incubation of lipopolysaccharide (LPS; Sigma‐Aldrich®, Merck, Shanghai, China, 1 μg/ml) for indicated time period. To examine the effects of IL‐4 (3 μg/ml; eBioscience®, ThermoFisher Scientific, Shanghai, China) and IL‐13 (3 μg/ml; eBioscience®, ThermoFisher Scientific, Shanghai, China) neutralizing antibodies, the two antibodies were added in medium at the same time with Rb1 (20 μM) for indicated period of time. To address the role of signal factor STAT6, cells were pre‐treated with 50 μM leflunomide (Enzo Life Sciences, Farmingdale, NY, USA), a STAT6 inhibitor, for 12 hrs before exposure to Rb1.

### Western blot analysis

Peritoneal macrophages were washed with ice‐cold PBS and homogenized on ice using RIPA lysis buffer (Beyotime) supplemented with complete protease inhibitor (Beyotime). Samples were loaded on 10% SDS‐PAGE and transferred onto PVDF membranes (Bio‐Rad, Shanghai, China). Membranes were blocked with 5% non‐fat dry milk and incubated with primary antibodies against iNOS (ab129372; Abcam, Cambridge, USA), Arg‐I (ab91279; Abcam), p‐STAT6 (ab54461; Abcam), STAT6 (ab32520; Abcam), respectively. β‐actin was used as control. The intensities of the corresponding protein bands were evaluated by densitometry *via* Image J (National Institutes of Health, USA) Protein expression was assessed relative to β‐actin or indicated protein.

### Flow cytometry

RAW264.7 cells were obtained from the American Type Culture Collection (ATCC, Manassas, VA, USA). Cells were cultured in Dulbecco's modified Eagle's medium (DMEM; Gibco®) with additional10% foetal calf‐serum (Gibco®) at 37°C humidified incubator under 5% CO2. RAW264.7 cells were incubated in the presence or absence of various concentrations of Rb1 that was always added 1 hr prior to LPS (1 μg/ml) treatment. RAW264.7 cells were washed, blocked and then incubated for 30 min. with APC‐conjugated anti‐mouse CD206 (141708; Biolegend, San Diego, CA, USA) using the appropriate isotype controls. The stained cells were acquired on a BD FACS Caliber flow cytometer (BD Biosciences, San Jose, CA, USA) and analysed with FlowJo v9.0 software (Tree Star, Inc., FlowJo v9.0, Ashland, Or, USA).

### Enzyme‐linked immunosorbent assays (ELISA)

Concentration of MMP‐9, IL‐10, IL‐4 and IL‐13 in culture supernatants was measured by ELISA kit (eBioscience®) following the manufacturer's protocol.

### Animal feeding and treatment

A total of 20 ApoE^−/−^ mice (male, 8 weeks old) were applied. The mice were obtained from Charles River Laboratories (Beijing, China). All ApoE^−/−^ mice were allowed to acclimatize for 1 week and fed a high‐fat diet (0.25% cholesterol and 15% cocoa butter) for 22 weeks. Then, mice were randomly divided into two groups (10 mice per group). In the Rb1 group, the mice were injected with Rb1 (50 mg/kg) daily for 7 weeks; and in the control group, mice were injected intraperitoneally with sterile PBS.

### Immunofluorescence and Immunohistochemistry

All the mice were euthanized. Hearts with attached aortic roots were harvested quickly frozen in OCT and sectioned (5 μM thick). Further immunohistochemical and immunofluorescent analyses were conducted as described below. Mouse aortic sinus cryosections were stained with anti‐inducible nitric oxide synthase antibody [iNOS] (ab178945; Abcam), anti‐Arginase antibody [Arg‐I] (ab91279; Abcam), anti‐alpha smooth muscle Actin antibody [SMA] (ab5694; Abcam) and anti‐Monocyte/Macrophage antibody [MOMA‐2] (MCA519G; AbD, UK), MMP‐9 (ab38898; Abcam). Morphometric analysis was performed on digitized images of intimal lesions and MOMA‐2‐immunostained areas using Image pro Plus software (Image‐Pro Plus, Version 6.0; Media Cybernetics, Houston, TX, USA). Plaque composition was assessed in cross sections of aortic root by immunostaining for MOMA‐2 (macrophage) and α‐smooth muscle actin (smooth muscle cell) and Sirius Red staining for collagen and Oil Red O for content of lipid. Collagen content of lesions was assessed with Sirius Red‐stained slides under polarizing light 17. Images were acquired on an inverted digital microscope system, and were processed by Image‐pro plus 6.0. For each slide, at least three HPF images were captured and evaluated in a blinded fashion. The percentage of the positive colour area with total area was calculated for each mouse. The plaque vulnerability index was calculated using the formula: vulnerability index=(lipid deposit%+macrophages%)(collagenfibres%+SMCs%) 18.

### Statistical analysis

All data were representative of at least three different experiments. Statistical analysis was performed using Graphpad Prism 6.0, La Jolla, CA, USA. The values were presented as mean ± S.D. Student's *t‐*test was used for two‐group comparisons. One‐way ANOVA was used for multi‐group comparisons following Turkey's test. Power analysis was applied to determine the difference of blood lipid between two groups. *P *<* *0.05 was considered significant.

## Results

### Rb1 promoted M2 macrophages polarization

**Figure 1:**
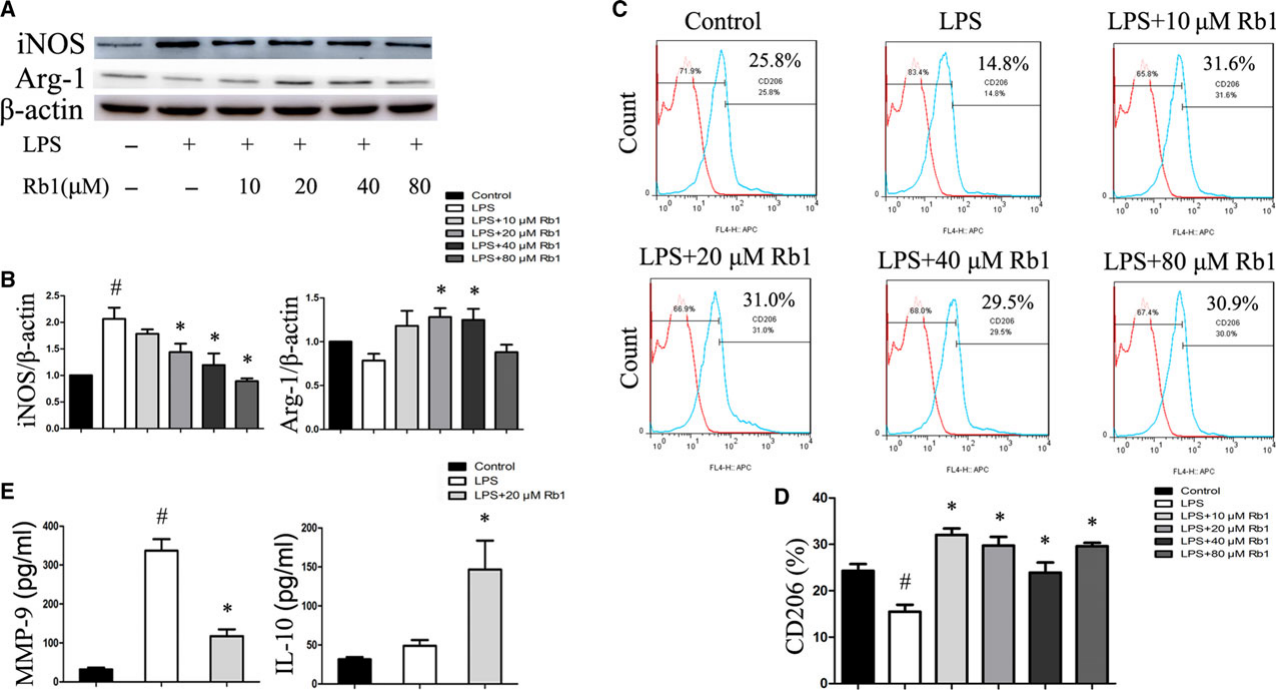
Rb1 induced macrophages M2 polarization. (**A**) Representative immunoblots of iNOS (M1 marker) and Arg‐I (M2 marker) in peritoneal macrophages treated with Rb1 at the different doses for 24 hrs. (**B**) iNOS and Arg‐I expression relative to the β‐actin level. (**C**) The expression of CD206 was examined by flow cytometry. (**D**) Percentages of RAW264.7 cells expressing of CD206. (**E**) ELISA for expression of pro‐inflammatory cytokine MMP‐9 and anti‐inflammatory cytokine IL‐10 in supernatant. Data are presented as means ± S.D.; *P <* *0.05, compared to LPS‐treated group; #*P *<* *0.05, compared to control group; *n *=* *3.

To explore the role of Rb1 in modulation of macrophage phenotype *in vitro*, the expression of iNOS (M1 marker) and Arg‐I (M2 marker) was detected in LPS‐stimulated peritoneal macrophages with or without treatment with Rb1. We found that Rb1 inhibited the expression of the iNOS in a dose‐dependent manner; meanwhile, Arg‐I level was increased at the dose of 20 and 40 μM, compared with the LPS‐stimulated macrophages (Fig. 1A and B), without increasing cell mortality with CCK‐8 test (Fig. S1A and B). In addition, the percentage of M2 phenotype (CD206^+^) cells of Rb1 group was much higher than that of the LPS‐stimulated group by flow cytometric analysis (Fig. 1C and D). Besides, we chose the dose of 20 μM in the following experiments.

Correspondingly, Rb1 suppressed the inflammatory response of macrophages induced by LPS as demonstrated by a significant decrease of pro‐inflammatory cytokine levels of MMP‐9 and an increase of anti‐inflammatory cytokines of IL‐10 (Fig. 1E). Therefore, these results suggested that Rb1 could skew macrophages to anti‐inflammatory M2 phenotype *in vitro*.

### IL‐4 and IL‐13 were involved in Rb1‐mediated macrophage polarization

**Figure 2:**
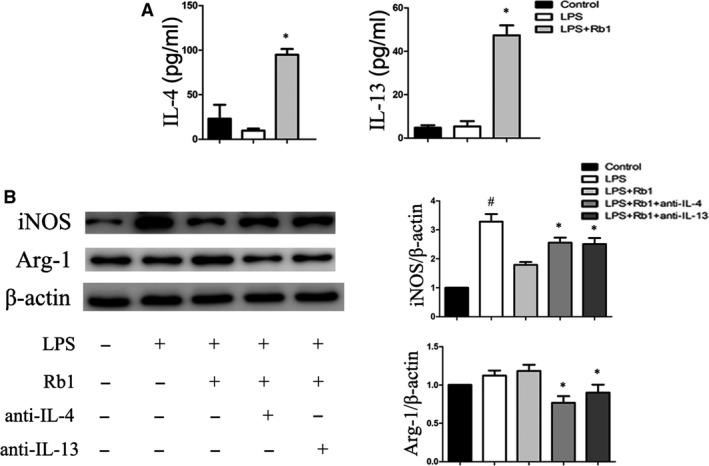
IL‐4 and IL‐13 involved in Rb1‐mediated macrophage polarization. (**A**) ELISA for IL‐4 and IL‐13 in supernatant of peritoneal macrophages treated with the indicated concentrations of Rb1 for 1 hr and then treated with LPS 1 μg/ml for 24 hrs. (**B**) Representative immunoblots of iNOS and Arg‐I protein in peritoneal macrophages treated by 20 μM Rb1 with or without IL‐4 and/or IL‐13 neutralizing antibody for 24 hrs. Data are presented as means ± S.D.; *P <* *0.05, compared to LPS‐treated group; #*P *<* *0.05, compared to control group; *n *=* *3.

IL‐4 and IL‐13 are known as the prototypical direct inducers of M2 macrophage 19, 20. To investigate whether Rb1 induced M2 macrophage polarization through increasing secreting IL‐4 and/or IL‐13, we measured IL‐4 and IL‐13 protein levels in supernatant. Our results showed that both the amount of IL‐4 and IL‐13 in supernatant was significantly increased after treatment with Rb1 for 24 hrs (Fig. 2A). These results indicated that Rb1 promoted IL‐4 and IL‐13 secretion in macrophages.

To further explore the role of IL‐4 and/or IL‐13 in Rb1‐induced M2 macrophage polarization, LPS‐stimulated macrophages were cultured in the presence or absence of neutralizing IL‐4 and/or IL‐13 antibodies, respectively. Notably, the effects of Rb1 on M2 macrophage polarization were attenuated with the treatment of IL‐4 or IL‐13 neutralizing antibodies (Fig. 2B). These results indicated that Rb1 induced M2 macrophage polarization partly through increasing secretion of IL‐4 and IL‐13.

### Rb1 induced M2 macrophage polarization through STAT6‐dependent pathway

**Figure 3:**
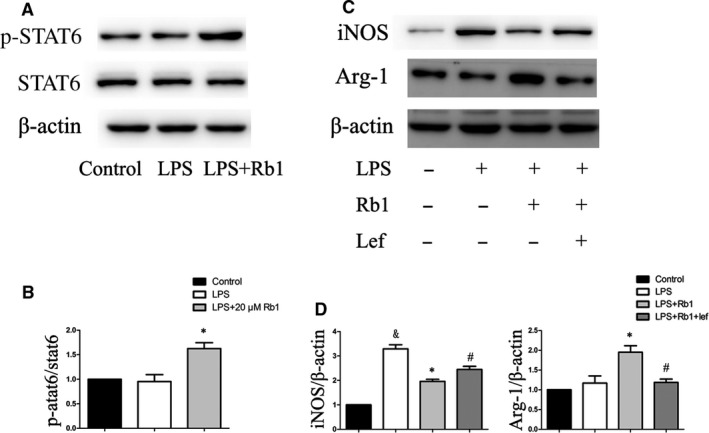
Rb1 induced macrophage M2 polarization through STAT6‐dependent pathway. (**A**) Representative immunoblots of the p‐STAT6 in peritoneal macrophages cells treated by Rb1. (**B**) Statistics of immunoblots presented as p‐STAT6 relative to the STAT6 level. (**C**) Representative immunoblots of the iNOS and Arg‐I protein in peritoneal macrophages cells treated by Rb1 for 24 hrs with or without the STAT6 inhibitor. (**D**) Statistics of iNOS and Arg‐I expression relative to the β‐actin level. Data are presented as means ± S.D.; *P <* *0.05, compared to LPS‐treated group; #*P *<* *0.05, compared to LPS+Rb1‐treated group; & *P *<* *0.05, compared to control group; *n *=* *3.

Previous studies showed that IL‐4 and IL‐13 induced M2 macrophages polarization through activation of signal transducer and activator of transcription factor 6 (STAT6) 17. To investigate whether STAT6 was involved in Rb1‐induced M2 macrophage polarization, we detected STAT6 phosphorylation level in mouse peritoneal macrophages after Rb1 treatment. The results showed that the level of p‐STAT6/STAT6 after treatment of 20 μM Rb1 was significantly higher than that in the LPS‐stimulated group (Fig. 3A and B). Furthermore, leflunomide, a STAT6 inhibitor, markedly reverted M2 macrophage polarization induced by Rb1 (Fig. 3C and D). These results indicated that Rb1 induced M2 macrophage polarization by producing IL‐4 and IL‐13 and through STAT6‐dependent pathway.

### Rb1 increased plaque stability in ApoE^−/−^ mice

**Figure 4:**
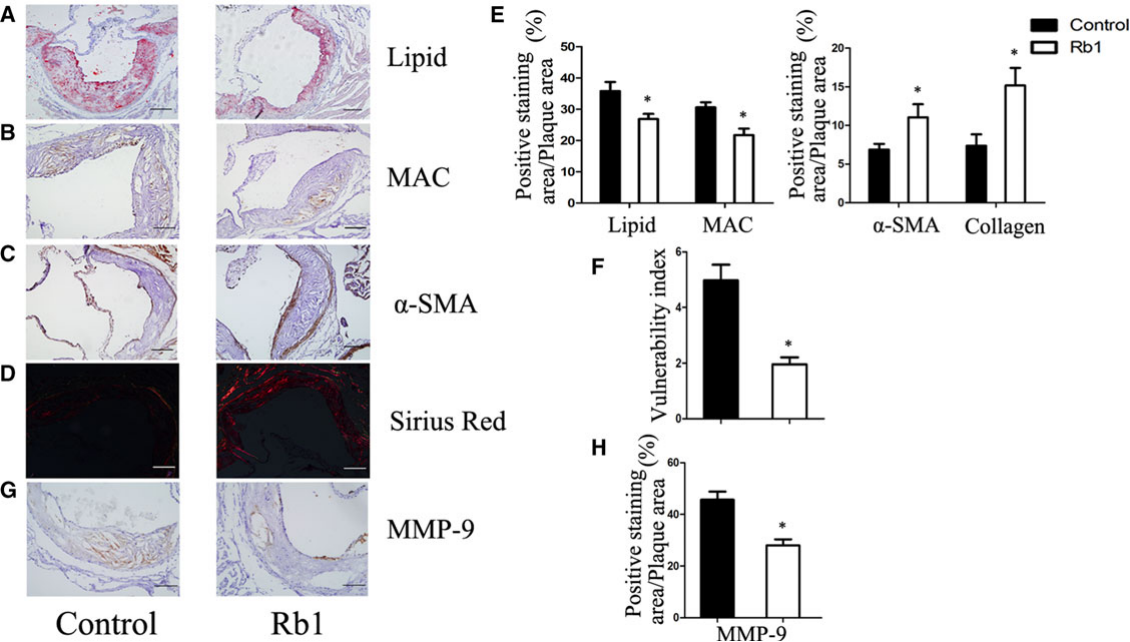
Rb1 obviously increased AS plaque stability of atherosclerotic ApoE^−/−^ mice. (**A**)(**B**)(**C**) Immnohistochemical staining of Oil red‐O staining of lipids, MOMA‐2 (macrophage marker), smooth muscle cells. (**D**) Representative images of Sirius Red‐staining for quantification of the lesion collagen content. (**E**) Statistics of (**A**)**–**(**D**) measured as area ratio. (**F**) Vulnerability index of plaque in corresponding groups calculated as (lipid deposit% + macrophages%) / (collagen fibres% + SMCs%). Data are means ± S.D.; Scale bar: 100 μm. *P <* *0.05; *n *=* *6. (**G**) (**H**) Representative immunohistochemistry and statistics of MMP‐9 expression. Quantification of the MMP‐9 positive staining area expressed as percentage of total lesion area. Data are presented as means ± S.D.; Scale bar: 20 μm. *P <* *0.05; *n *=* *6.

As we found Rb1 promoted M2 macrophages polarization *in vitro*, we next validated the effect of Rb1 on arterial inflammation and atherosclerotic plaque stability *in vivo*. Atherosclerotic vulnerable plaque is characterized by a mass of macrophage infiltration and lipid accumulation, as well as a thin cap with less collagen and smooth muscle cell (SMCs) 21, 22. Our results showed that the morphology of plaque in control group coincides with the feature of typical vulnerable plaque. Composition of plaque has obviously changed after Rb1 treatment, in which the content of lipids and macrophages was significantly decreased, but that of collagen and SMCs was significantly increased after treatment with Rb1 (Fig. 4A–E). Consequently, the vulnerability index of plaque in Rb1‐treated mice was significantly reduced compared to the control group (Fig. 4F). Besides, staining for matrix degrading proteases‐9 (MMP‐9), a pro‐atherosclerosis cytokine secreted by macrophages, was also decreased significantly in atherosclerotic lesions of mice in the Rb1 group (Fig. 4G and H). These results indicated that Rb1 treatment enhanced atherosclerotic plaque stability.

We have tested respective components of blood lipid in both groups (**Table** S1). Although there was a tendency of lower LDL‐c, HDL‐c and triglyceride in Rb1 treatment group, there was no significant difference between two groups. A post hoc power analysis has been conducted and the power of test is weak. Therefore, we cannot clearly conclude that Rb1 has definite effects on blood lipid so far.

### Rb1 treatment promoted M2 macrophage polarization in atherosclerotic plaque

**Figure 5:**
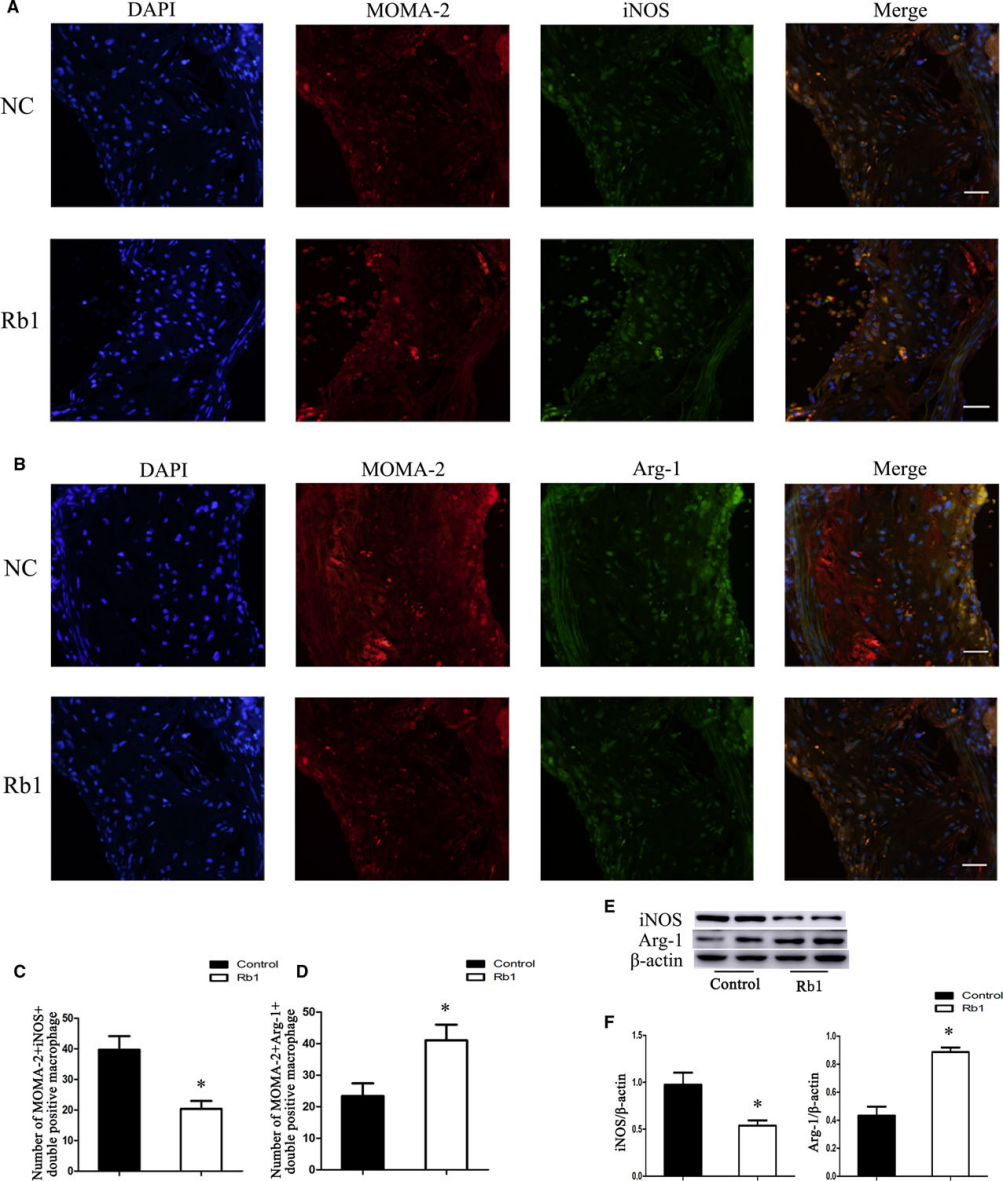
Effects of Rb1 on macrophage polarization in atherosclerotic lesions of ApoE^−/−^ mice. (**A**,** B**) Representative images of MOMA‐2^+^ iNOS ^+^ and MOMA‐2^+^Arg‐I^+^ macrophages *in situ* in corresponding groups (NC: control group; Rb1: Rb1 treatment group). (**C, D**) Statistics of the number of MOMA‐2^+^ iNOS ^+^ and MOMA‐2^+^Arg‐I^+^ macrophages in atherosclerotic lesions in control and Rb1‐treated ApoE^−/−^ mice. Scale bar: 20 μm. *P <* *0.05; *n *=* *6. (**E**) Representative immunoblots of iNOS (M1 marker) and Arg‐I (M2 marker) *in vivo*. (**F**) Statistics of iNOS and Arg‐I expression relative to the β‐actin level. Data are presented as means ± S.D.; *P <* *0.05, compared to control group; *n *=* *3.

To investigate whether macrophage polarization switch was involved in the role of Rb1 on atherosclerosis, immunofluorescent analysis of iNOS and Arg‐I expression was applied to identify and evaluate, respectively, M1 and M2 macrophage (MOMA‐2 ^+^ cells) in aortic root cryosections from Rb1 and control group. The number of MOMA‐2^+^ iNOS^+^ double positive macrophages (M1 macrophage) in atherosclerotic lesions was significantly decreased in the Rb1 group compared with the control group (Fig. 5A–D). Meanwhile, the number of double positive MOMA‐2^+^Arg‐I^+^ macrophages (M2 macrophage) was significantly increased in the atherosclerotic lesions of Rb1 group. Similar results were observed at the protein level of iNOS and Arg‐I in aortic lysates of both groups by Western blotting (Fig. 5E and F). These results suggested that Rb1 treatment skewed macrophages to M2 phenotype in atherosclerotic lesions.

## Discussion

In this study, we confirmed that Rb1 promoted M2 macrophage polarization and restricted inflammatory response, which further contributed to enhance atherosclerotic plaque stability in ApoE^−/−^ mice. The effects of Rb1 on macrophage M2 polarization were majorly achieved by increasing the production of IL‐4 and IL‐13 and successive STAT6 phosphorylation.

In recent decades, CAD has caused growing disability and mortality and has become one of the major health burdens 1. Atherosclerosis is the major pathologic basis of CAD. Since Framingham heart study, we have stepped into an era to fight against with atherosclerosis. We have experienced great revolution in AS treatment, from Aspirin to statins, from medicine to stent, from treatment to prevention. Although the integrated treatment shows effective so far, there are still many serious problems we must face. Inevitable progression of AS plaque even under current therapy becomes one of the challenges, in which condition the plaque will become vulnerable and easy to rupture, causing acute coronary syndrome 1. Therefore, approaches aiming at stabilizing and retarding the AS plaque have manifested effective and may become promising therapies 23, 24, 25.

With the AS progression, the plaques exhibit advanced features, with lipid accumulation, macrophages infiltration, covered with fibrous cap and smooth muscle cells 17, 21. Studies have well demonstrated that balance of macrophage phenotypes M1 and M2 exerts potential effect on regulating AS plaque stability, with M1 leading to vulnerable plaque while M2 leading to stable plaque 26. Therefore, strategies trying to promote M2 macrophage polarization may be available to stabilize plaques and limit AS progression 27, 28.

Traditional herbal medicine inspired us with newly discovered extracts. Ginsenoside Rb1 is a representative component of ginseng, an herbal medicine widely used in Eastern Asia. Previous studies have demonstrated its multifarious pharmacological effects, including anti‐inflammation, anti‐obesity and improving immunity 13, 14. Recently, several studies have detected the effects of Rb1 in the cardiovascular system. Zheng and Cui showed, respectively, that Rb1 protected against cardiac remodelling and ischaemia/reperfusion‐induced myocardial injury, and Lan reported Rb1 could prevent homocysteine‐induced endothelial dysfunction 13, 14, 15. However, little is known about the role of Rb1 on atherosclerotic plaque stability.

Given the role of macrophage polarization in inflammatory response, we first investigated the effects of Rb1 on macrophage polarization. Our research verified that Rb1 induced a phenotypic alteration of macrophages from M2 to M1 *in vitro*, with consequential reduced pro‐inflammatory cytokine (MMP‐9) and enhanced anti‐inflammatory cytokine (IL‐10). These data suggested that Rb1 might alleviate inflammation by inducing M2 macrophages polarization.

Having proved that Rb1 could alleviate inflammation by skewing M2 macrophages polarization, we next determined the vascular‐protective effect of Rb1. In the AS lesion from Rb1 treatment group, we observed a reduction of lipids accumulation and macrophage infiltration, as well as an increase of collagen and SMCs, which indicated a reduced vulnerability index of plaque. Consistent with *in vitro*, staining for MMP‐9 was also decreased significantly in atherosclerotic lesions of mouse in the Rb1 group. To explore the mechanism by which Rb1 protected against atherosclerosis, we next investigated the M1/M2 macrophage polarization in atherosclerotic lesions. Not surprisingly, treatment with Rb1 promoted M2 macrophage polarization in atherosclerotic plaque. These results suggested that Rb1 treatment could increase AS plaque stability by promoting M2 macrophage polarization.

We further explored the underlying mechanisms by which Rb1 facilitated M2 macrophages polarization *in vitro*. IL‐4 and IL‐13 have been well demonstrated as classical and powerful M2 macrophage cytokines 19, 20. The pathways of the cytokines converge on signal transducer and activator of transcription 6 (STAT6) 26, which is critical for relative gene transcription and is considered as a potential new target in human atherosclerotic disease 29. In our study, we found that Rb1 could effectively elevate both IL‐4 and IL‐13 secretion and promote STAT6 phosphorylation consecutively. This effect could be abrogated *via* adding either neutralizing antibodies or STAT6 inhibitor. Therefore, we concluded that Rb1 promoted M2 macrophage polarization by enhancing IL‐4 and IL‐13 secretion and through STAT6‐dependent pathway.

There remains limitation in our work. It is reported that change in balance of blood lipids may affect macrophage polarization. In our work, although we did not reach a definite role of Rb1 on blood lipid, we manifested that Rb1 could regulate macrophage polarization directly *in vitro*. Till now, there is no consensus about Rb1 on blood lipid 30, 31, and it is promising to be further investigated.

Taken together, ginsenoside Rb1 protected against atherosclerosis by promoting M2 macrophage polarization and limiting intraplaque inflammatory response, which was achieved by increasing the production of IL‐4 and IL‐13 and STAT6 phosphorylation. Our study provides new experimental evidence for possibility of Rb1 in prevention and treatment of atherosclerosis.

## Conflict of interest

The authors declare no conflicts of interest.

## Supporting information


**Fig. S1** Effect of Rb1 on macrophages cell viability
**Table S1** Effect of Rb1 on serum lipid profiles of ApoE^−/−^ mice
