# Corrigendum

**DOI:** 10.1111/jcmm.17887

**Published:** 2023-09-01

In Zhang et al.,^1^ the published article contains errors in Figure 5A,B. The correct figures are shown below. The authors confirm all results and conclusions of this article remain unchanged.

**FIGURE 5:**
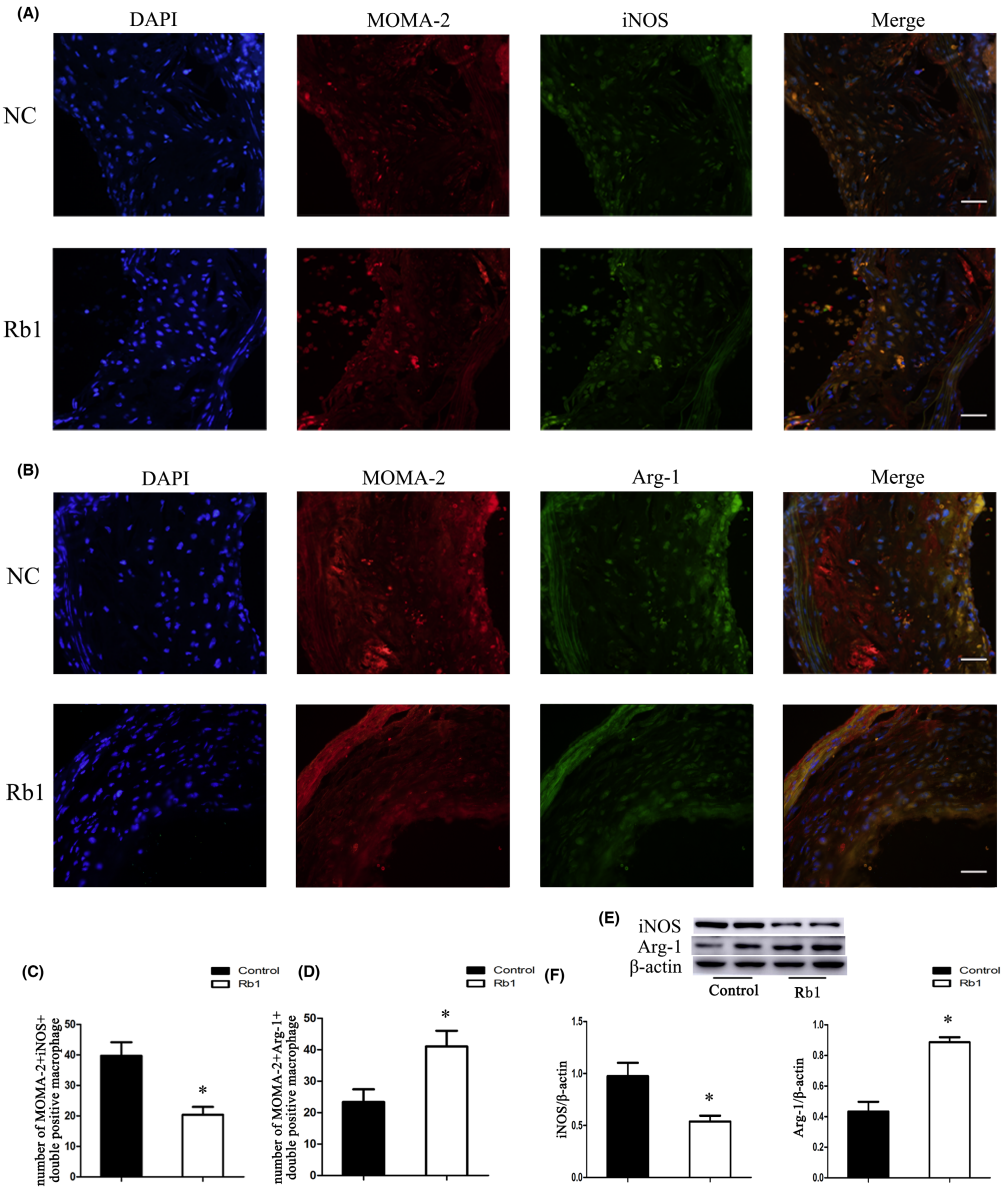
Effects of Rb1 on macrophage polarisation in atherosclerotic lesions of ApoE^−/−^mice. (A, B) Representative images of MOMA‐2^+^iNOS^+^ and MOMA‐2^+^Arg‐1^+^ macrophages in situ in control group (NC) and Rb1 treatment group (Rb1). (C, D) Statistics of the number of MOMA‐2^+^iNOS^+^ and MOMA‐2^+^Arg‐1^+^ macrophages in atherosclerotic lesions in control group (NC) and Rb1 treatment group (Rb1). Scale bar: 20 μm. *p < 0.05; *n* = 6. (E) Representative immunoblots of iNOS (M1 marker) and Arg‐1 (M2 marker) in vivo. (F) Statistics of iNOS and Arg‐1 expression relative to the β‐actin level. Data were presented as means ± S.D.; *p < 0.05, compared to control group; *n* = 3.

The authors apologize for the inconvenience this may cause.
