# Supplementary material for: Ginsenoside Rb1 enhances atherosclerotic plaque stability by skewing macrophages to the M2 phenotype
Source: J Cell Mol Med. 2017 Sep 25;22(1):409–16. doi: 10.1111/jcmm.13329 (PMC5742675; doi:10.1111/jcmm.13329)
Supplement: Supplementary file 1 — Fig. S1 Effect of Rb1 on macrophages cell viability Table S1 Effect of Rb1 on serum lipid profiles of ApoE−/− mice [file JCMM-22-409-s001.doc]

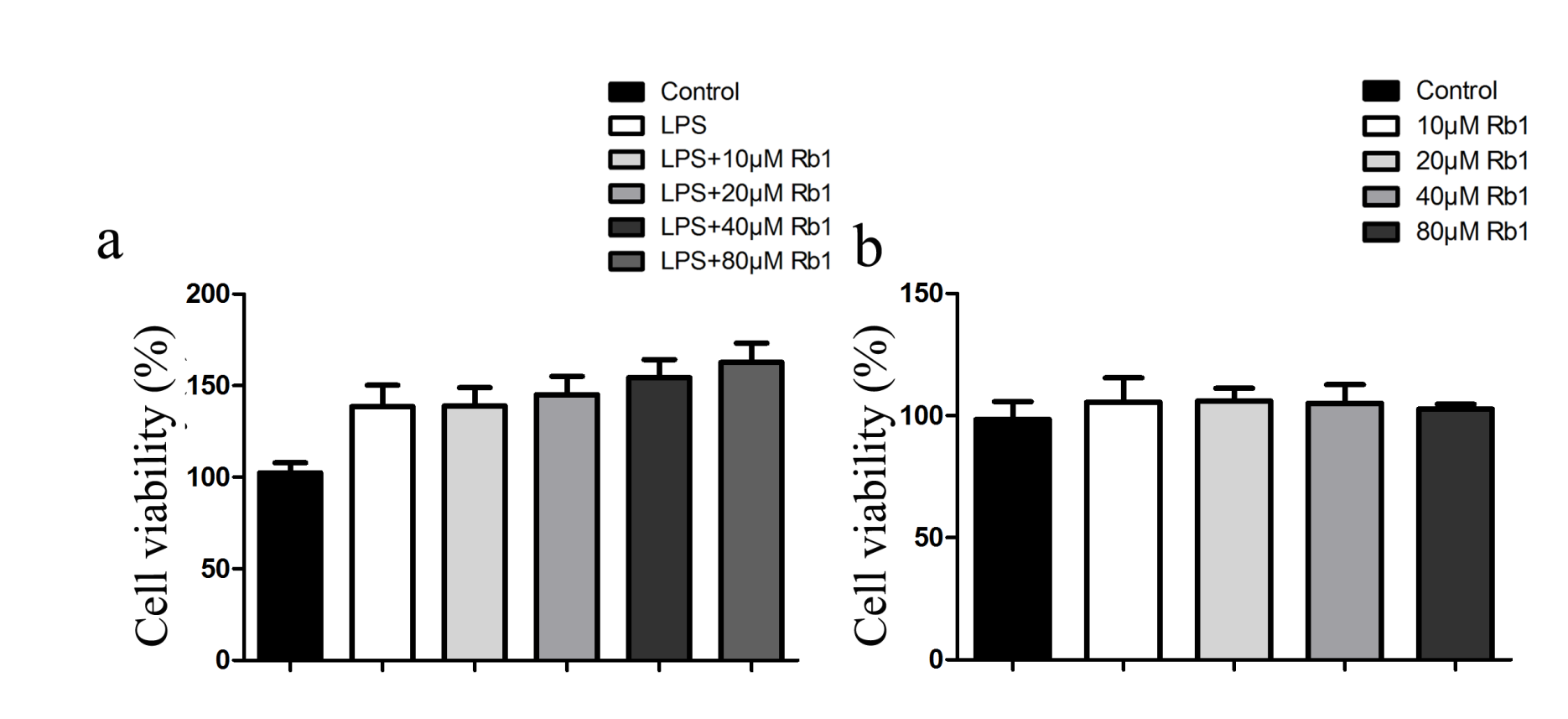


**Figure S1**: Cytotoxicity of macrophages. Macrophages induced either with (a) or without (b) LPS were treated with indicated concentrations of Rb1 for 24 h. The cell viability of each condition was analyzed using CCK8 assay. The relative cytotoxicity is calculated as relative OD values to untreated cell. Data are analyzed by one-way ANOVA and presented as the means ± SD of three independent experiments.

**Table S1: Effect of Rb1 on serum lipid profiles of ApoE^-/-^** mice.

| **Groups** | **TC**  **(mmol/L)** | **TG**  **(mmol/L)** | **HDL-C (mmol/L)** | **LDL-C (mmol/L)** |
| --- | --- | --- | --- | --- |
| **Control** | **19.26±0.41** | **2.28±0.41** | **7.02±0.62** | **4.23±0.58** |
| **Rb1** | **19.30±0.54** | **2.09±0.24** | **6.93±0.74** | **3.66±0.86** |
| ***P*** | **ns** | **ns** | **ns** | **ns** |

Data are expressed as mean±SD; TC: total cholesterol; TG: triglyceride; HDL-C: high-density lipoprotein; LDL-C: low-density lipoprotein; ns: not significant; n=6
